# Distinct physical activity and sedentary behavior trajectories in older adults during participation in a physical activity intervention: a latent class growth analysis

**DOI:** 10.1186/s11556-021-00281-x

**Published:** 2022-01-05

**Authors:** Tiara Ratz, Claudia R. Pischke, Claudia Voelcker-Rehage, Sonia Lippke

**Affiliations:** 1 Department of Psychology & Methods, Jacobs University Bremen, Bremen, Germany; 2 Institute of Medical Sociology, Centre for Health and Society, Medical Faculty, Heinrich Heine University Duesseldorf, Duesseldorf, Germany; 3 Department of Neuromotor Behavior and Exercise, Institute of Sport and Exercise Sciences, University of Muenster, Muenster, Germany

**Keywords:** Exercise, Sitting, Trajectory, Mixture modeling, Health behavior change

## Abstract

**Background:**

This study aimed to identify latent moderate-to-vigorous intensity physical activity (MVPA) and sedentary behavior (SB) trajectories in older adults participating in a randomized intervention trial and to explore associations with baseline social-cognitive predictors.

**Methods:**

Data were assessed at baseline (T0, participants were inactive or had recently become active), after a ten-week physical activity intervention (T1), and a second 24-week intervention phase (T2). Latent class growth analysis was used on accelerometer-assessed weekly MVPA and daily SB, respectively (*n* = 215 eligible participants). Activity changes within trajectory classes and baseline social-cognitive predictor differences between trajectory classes were analyzed.

**Results:**

A “stable insufficient MVPA” (*n* = 197, *p* for difference in MVPA level at T0 and T2 (*p*_T0-T2_) = .789, effect size (Cohen’s *d*) = .03) and a “stable high MVPA” trajectory (*n* = 18, *p*_T0-T2_ = .137, *d* = .39), as well as a “slightly decreasing high SB” (*n* = 63, *p* for difference in SB (*p*_T0-T2_) = .022, *d* = .36) and a “slightly increasing moderate SB” trajectory (*n* = 152, *p*_T0-T2_ = .019, *d* = .27) emerged. Belonging to the “stable high MVPA” trajectory was associated with higher action planning levels compared to the “stable insufficient MVPA” trajectory (*M* = 5.46 versus 4.40, *d* = .50). Belonging to the “decreasing high SB” trajectory was associated with higher action self-efficacy levels compared to the “increasing moderate SB” trajectory (*M* = 5.27 versus 4.72, *d* = .33).

**Conclusions:**

Change occurred heterogeneously in latent (not directly observed) subgroups, with significant positive trajectories only observed in the highly sedentary.

**Trial registration:**

German Registry of Clinical Trials, DRKS00016073, Registered 10 January 2019.

**Supplementary Information:**

The online version contains supplementary material available at 10.1186/s11556-021-00281-x.

## Background

If every inactive individual became as physically active as recommended by the World Health Organization [1], this elimination of physical inactivity could lead to a gain of roughly half a year in life expectancy and to a reduction of all-cause mortality by 7.5% in Germany [2]. Yet, Germany belonged to the five countries with the largest increases in the prevalence of physical inactivity from 2001 to 2016 among 65 countries worldwide [3]. The prevalence of sedentary behavior (SB) among Germans increased from 50 to 53.7% between the years 2002 and 2017 [4]. The uptake of physical activity even in old age is beneficial to health [5, 6]. Physical activity interventions targeting older adults have been shown to be effective [7, 8], but evidence regarding their effectiveness for behavior change maintenance is inconclusive [9, 10]. This article’s objective is to investigate *heterogenous change trajectories* in German older adults as a potential cause for inconclusive results regarding behavior change maintenance.

In health behavior change interventions, not every individual might follow the same change trajectory and the utility of particular behavior change techniques may vary across individuals. Theories such as the transtheoretical model [11, 12] and the health action process approach (HAPA) [13, 14] suggest that individuals with differing preconditions or characteristics move differently through stages of behavior change. These characteristics are known as *social-cognitive predictors*. Participants of physical activity intervention studies may consist of *subgroups*. For example, some may experience an increasing change trajectory and demonstrate high levels of maintenance self-efficacy (the perceived capacity of overcoming barriers to perform physical activity) or action planning (the ability to identify cues relating to when, where and how to be physically active). Others might keep their physical activity level constant or become less active and possibly demonstrate low levels of action self-efficacy (the perceived capacity of performing physical activity) [15]. Examining the existence of *latent* (i.e., unobserved) change trajectory subgroups could improve the understanding of heterogeneous behavior change occurring in interventions. This knowledge may assist future studies in more targeted recruitment efforts and in identifying components of behavior change interventions required to improve effectiveness and maintenance, especially for subgroups belonging to low or decreasing physical activity trajectories [16].

The analysis technique *longitudinal mixture modeling* aims to identify latent homogenous subgroups with similar change or trajectory patterns [17, 18]. Studies adopting this so-called person-centered approach show that individuals differ in their *long-term* physical activity change trajectories [19–21]. That is, over the course of multiple years, distinct change trajectories can be observed. However, the evidence on differing *short-term* physical activity change trajectories in intervention studies spanning over a maximum of one year is scarce. A study on physical activity promotion in the office-setting over the course of one year identified three distinct change trajectories: a decrease from a high level, a stable moderate level, and an increase from a low level of physical activity [16]. A recent study on young adults participating in a physical activity intervention trial reports four distinct trajectories over the course of one year, which they labeled normal/decrease, normal/increase, normal/rapid increase, and high/increase [22]. In the early 2010’s, researchers applied health behavior change theories to the prediction of latent physical activity trajectories and found associations with social-cognitive predictors [20, 23]. To the best of the authors’ knowledge, an investigation of latent short-term trajectories and associations with social-cognitive predictors in physical activity interventions was not performed for older adults, yet.

The objective of this study was to investigate latent moderate-to-vigorous intensity physical activity (MVPA) trajectories and associated factors in older adults participating in a nine-month physical activity intervention trial. The secondary objective of this study was to identify and investigate latent change trajectories regarding SB. It was hypothesized that 1) there are *latent subgroups* which differ by their MVPA and SB *trajectory* over the course of the nine-month intervention period; and 2) latent *class membership* is associated with baseline social-cognitive predictors for physical activity behavior change.

## Methods

### Procedure and participants

This study belongs to the Physical Activity and Health Equity: Primary Prevention for Healthy Ageing (AEQUIPA) prevention research network [24] and uses data obtained in the second study phase of the PROMOTE-study [25]. The primary aim of the second study phase was to compare the effectiveness of two different physical activity intervention modalities (web- vs. print-based intervention) on changes in physical activity among older adults. Ethical approval for the study was obtained on July 3rd, 2018, from the Medical Association in Bremen. All study participants were fully informed about the study and provided informed consent. The data analyses reported in this paper are of exploratory nature.

A random sample of *n* = 3492 adults aged 60 years and above residing in Bremen, Northwestern Germany, was drawn from the residents’ registration office and contacted via mail. Additionally, press releases and public talks were used to recruit study participants who could contact the study team and choose to participate after receiving further information on the study. Older adults were included if they provided informed consent and were either inactive or recently active, meaning that they had not been sufficiently physically active for more than one year. Individuals with time and health constraints, as well as those not owning a mobile phone or not being able to use it regularly, were excluded. Further details on eligibility criteria were published in the study protocol [25]. The final baseline study sample consisted of *n* = 242 individuals (see Additional file 1 for the flow chart). Eligible older adults were randomly assigned to a print-based intervention or a web-based intervention during a telephone interview with a study nurse. The intervention groups were assigned to alternating weeks. During the telephone interview, participants were randomized by having them choose a weekly appointment while being blinded to the intervention condition assigned to the chosen week.

The print-based intervention group (*n* = 113) received physical activity recommendations based on the World Health Organization guidelines [1], a printed physical activity diary and a brochure with age-appropriate exercises. The web-based intervention group (*n* = 129) received the same program in the form of a website and an android smartphone-application. The interventions were designed to promote self-monitoring of physical activity, were based on health behavior change theory [14, 26] and incorporated behavior change techniques [27]. A subgroup (30% of the web-based intervention group, *n* = 38) additionally received an activity tracker (Fitbit Zip, Fitbit, San Francisco, USA), substituting the subjective self-monitoring intervention with an objective self-monitoring component. The interventions were mainly home-based but included face-to-face components. In the first intervention phase, each individual was offered to participate in ten weekly group sessions, covering 60 min of exercise training and 30 min of health education. During the second intervention phase lasting six months, four health education group sessions were offered. Older adults were not blinded to group affiliation once they were assigned to it, and neither were investigators.

Participants completed a self-administered questionnaire and wore an accelerometer for seven days during waking hours on their right hip at baseline (T0, January to April 2019), at the first follow-up (T1, April to July 2019) and at the second follow-up (T2, September 2019 to January 2020). A cognitive screening test was conducted during the first weekly group session. The dropout rate after T2 completion was 33.9% (see Additional file 1 for numbers per intervention group regarding loss to follow-up).

### Measures

#### Physical activity and sedentary behavior

Physical activity and sitting time were assessed using accelerometers (GT3X+, ActiGraph, Pensacola, USA). Valid days were identified using the Actilife 6.8.0 software and R 3.6.1. Valid days were defined as having ≥8 h of valid wear-time, with no definition of maximum wear-time. Participants needed to have at least three valid days, including one weekend day. Total minutes of light (0–2690 counts per minute), moderate (2691–6166 counts per minute), and vigorous physical activity (6167–9642 counts per minute), as well as sitting time (0–99 counts per minute) were derived by using one-second epochs for the categorization of counts per minute according to cut-off values considering the vector magnitude [28]. Minutes per week were derived by dividing the total minutes spent in light, moderate or vigorous physical activity, respectively, by the days the accelerometer was worn and then multiplying the value by seven. Additionally, MVPA was derived using 2691–9642 counts per minute and counting only the time spent in bouts of at least ten minutes according to the physical activity recommendations given in the study. The average minutes spent with SB per day were calculated by dividing the total minutes spent with SB in bouts of at least 30 min by the number of the days the accelerometer was worn.

#### Baseline measures

Demographic information, including sex and date of birth, was assessed as reported in the study protocol [25]. The International Standard of Education (ISCED) [29] was used to code an educational status score, which was dichotomized into “low/moderate” and “high” educational status. Need-weighted income per capita was derived according to the German Microcensus [30] and tertiled into “low”, “moderate” and “high”. Employment was dichotomized into “fully retired” and “other than fully retired”. Body mass index was calculated from self-reported weight and height and dichotomized into “underweight/normal weight” and “overweight/obese”. Cognitive screening was administered using the Mini Mental State Examination 2 - brief version (MMSE-2-BV) [31, 32].

Social-cognitive predictors for engaging in the recommended levels of physical activity were assessed using validated measures as reported in the study protocol [25] and published results of the first study phase [33]. Older adults were asked to rate respective statements on Likert-scales from 1 (= totally disagree) to 7 (= totally agree). For example, intention was assessed with one item which consisted of the statement “I intend to engage in moderate endurance training for at least 150 minutes per week (not tiring, slightly sweating) and strength and balance training twice a week.” Furthermore, the following social-cognitive predictors were assessed: positive and negative outcome expectations (two items, respectively), self-efficacy (one item measuring action self-efficacy, two items measuring maintenance self-efficacy, and two items measuring recovery self-efficacy), action and coping planning (three items, respectively), and habit strength (two items). A detailed description of the assessed social-cognitive predictors has been provided in previous publications [33]. Mean scores were aggregated per social-cognitive predictor (Cronbach’s alpha ranged from .72 to .96) except for negative outcome expectations (Cronbach’s alpha = .65).

### Outcome and analysis sample definition

The primary outcome was minutes of MVPA in bouts of at least ten minutes per week, in line with the physical activity recommendations given to study participants. The secondary outcome was the average minutes spent sitting in at least 30-min bouts per day. Subgroups were not defined a-priori as this study’s objective was the identification of unobserved subgroups in terms of latent trajectories (not directly observed). However, based on a systematic review on physical activity trajectories [21], the maximum possible number of trajectory classes was set to six, possibly including the following categories: increasing, stable high, stable sufficient, decreasing moderate, stable insufficient, and decreasing low physical activity.

Older adults were included in the analysis sample if they were cognitively healthy (MMSE-2-BV ≥ 13) and had existing values for the primary outcome on at least one timepoint. The inclusion value for the MMSE-2-BV was ≥15 originally, but it was changed to ≥13 based on previous studies [34, 35]. The analyzed sample (*n* = 215, see Additional file 1) did not differ from the recruited sample, which was tested considering a set of socio-demographic, psychological and health-related characteristics (effect sizes were all < .20).

### Statistical analyses

#### Latent trajectory analysis strategy

Finite mixture models were calculated using an expectation-maximization algorithm for maximum likelihood estimation of model parameters in Mplus version 8.4 [36]. The best-fitting latent trajectory model was determined following the steps proposed by van der Nest et al. [17], and the recommendations provided by the Guidelines for Reporting on Latent Trajectory Studies (GRoLTS) Checklist [37]. The slopes for the three timepoints were set to be 0, 2.66 and 8.35 – according to the median months the measurements lay apart. Latent class growth analysis (LCGA) was conducted to identify latent MVPA and SB trajectories, respectively. LCGA for SB was adjusted for wear-time, as the amount of time the accelerometer was worn correlated with SB.

Investigated fit indices to determine LCGA model fit were the Bayesian Information Criterion (BIC), Akaike’s Information Criterion (AIC), and sample-size adjusted BIC (SABIC). An elbow plot of fit indices was created to visualize the point at which the decrease in fit indices became less in extent. The *p*-values of the Lo-Mendell-Rubin likelihood ratio test (LMR-LRT) and the bootstrapped likelihood ratio test (BLRT) were considered to determine whether the respective model provided a better fit than the model with one class less. To validate the selected model, the minimum class size was evaluated with the cut-off at 5% and an entropy approaching 1.000 indicating higher certainty. The selected model was critically reviewed for clinical and theoretical plausibility and meaningfulness. The models were rerun using different starting values to ensure that the estimation did not result in local maxima. The dataset including the categorical variable indicating the latent trajectory class was exported to SPSS 26 (IBM Corp. Released 2019. IBM SPSS Statistics for Windows, Version 26.0. Armonk, NY: IBM Corp) to investigate changes within latent trajectory classes and associations with baseline social-cognitive predictors.

#### Changes by Timepoint and activity-type and associations with social-cognitive predictors

To analyze whether a linear function could describe the data well, changes within the latent trajectory classes between the three timepoints were investigated using paired samples t-tests. Changes in MVPA, SB, light, moderate and vigorous physical activity were analyzed. Associations of latent trajectory class membership with social-cognitive predictors were investigated with independent samples t-tests. An investigation of social-cognitive indicators as predictors of latent trajectory class membership in logistic regression was deliberately omitted. Calculating odds ratios for social-cognitive indicators would provide information on the likelihood of belonging to a latent change trajectory given a one-unit increase in a social-cognitive indicator. Comparing the mean values between groups and testing for statistical significance between them, on the other hand, was deemed more relevant and more suitable with the aims of this manuscript.

#### Missing data handling and interpretation of effects

Finite mixture models were calculated using full-maximum likelihood estimation, as missing value analysis indicated that the precondition of data missing at random was met. For analyses of changes within latent trajectory classes and associations with baseline variables, missing values were imputed using multiple imputation with predictive mean matching. For imputed data, the mean and standard error (*SE*) were calculated to report continuous indicators by latent trajectory classes for each assessment timepoint. Cohen’s *d* was calculated as a measure of effect size based on the pooled mean differences and standard deviations and Cramer’s *V* was averaged across all datasets. All analyses were carried out under the intention-to-treat assumption. We would like to stress that the analyses of this exploratory study did not serve to evaluate intervention effectiveness by comparing web- and print-based components of the physical activity intervention. These primary outcomes are reported elsewhere [38]. As primary outcome analyses showed that there was no substantial difference between the intervention groups in terms of effects on MVPA or SB [38], the analyses reported here considered all intervention groups as a joint group under the assumption of no differential effect present between the intervention groups.

## Results

### Latent trajectory analyses

#### Physical activity

The estimated mean minutes of MVPA per week in the initial growth curve model assuming just one latent change trajectory were *M*_T0_ = 83.45, *M*_T1_ = 81.90 and *M*_T2_ = 75.13. This slight downward trend in MVPA in the whole study sample has been discussed elsewhere [38].

A spaghetti plot displaying individual MVPA trajectories indicated some degree of variation in the trajectories, meaning the presence of underlying subgroups (data not shown). Therefore, the investigation of latent subgroups using LCGA was continued. The elbow plot (Additional file 2) suggested that the decrease in fit indices became less steep after two classes. The BLRT *p*-value, however, remained significant. This phenomenon has been previously reported to occur in empirical studies [37]. However, the smallest class contained less than 5% in the three-class solution. Thus, no further classes were added to the model. Based on the elbow plot (Additional file 2), an entropy of .949 (Table 1) and high classification probabilities (Table 2), the two-class model was chosen.

**Table 1:** Fit Statistics for the Latent Class Growth Analysis of Change Trajectories

Class	Log likelihood	AIC	BIC	SABIC	Entropy	smallest class %	LMR-LRT	BLRT
Moderate-to-vigorous physical activity								
1	− 2949	5908	5925	5909	1	–	–	–
**2**	**− 2872**	**5760**	**5787**	**5762**	**0.949**	**8.37**	**.071**	**<.001**
3	− 2846	5715	5752	5717	0.885	4.19	.741	<.001
Sedentary behavior								
1	− 2963	5941	5965	5943	1	–	–	–
**2**	**− 2907**	**5836**	**5873**	**5838**	**0.787**	**29.30**	**0.005**	**<.001**
3	− 2877	5785	5836	5788	0.804	3.26	0.273	<.001

*Note.* Classes were consecutively added until the best fitting model was identified. The selected models are in boldface

*AIC* Akaike’s information criterion, *BIC* Bayesian information criterion, *SABIC* sample size adjusted BIC, *LMR-LRT p*-value of Lo-Mendell-Rubin adjusted likelihood ratio test, *BLRT p*-value of bootstrap likelihood ratio test

**Table 2:** Numbers, Proportions and Posterior Probabilities for the Latent Trajectory Classes

Latent Trajectory Class	Number (%)	Posterior Probabilities	
		Class 1	Class 2
Moderate-to-vigorous physical activity			
Stable high MVPA	18 (8.37)	0.924	0.076
Stable insufficient MVPA	197 (91.63)	0.007	0.993
Sedentary behavior			
Increasing moderate SB	152 (70.70)	0.965	0.035
Decreasing high SB	63 (29.30)	0.119	0.881

*Note. MVPA* moderate-to-vigorous physical activity, *SB* sedentary behavior

The sample was comprised of a “stable insufficient MVPA” class and a “stable high MVPA” class. The “stable insufficient MVPA” class consisted of *n* = 197 individuals with weekly mean (*SE*) MVPA = 59.23 (5.30) minutes at T0, 67.05 (6.34) minutes at T1 and 61.55 (7.64) minutes at T2. There was little, nonsignificant variation in MVPA between the timepoints and effect sizes were very small (*p*_T0-T1_ = .239, *d* = .09; *p*_T1-T2_ = .517, *d* = .06; *p*_T0-T2_ = .789, *d* = .03). Thus, the trajectory was labeled as stable over time at an insufficient level.

The “stable high MVPA” class consisted of *n* = 18 individuals with weekly mean (*SE*) MVPA = 348.55 (39.01) minutes at T0, 254.78 (50.36) minutes at T1 and 245.29 (49.13) minutes at T2. The mean difference between T0 and T2 seemed large with roughly 100 min, yet the difference did not reach statistical significance and effect sizes were small (*p*_T0-T1_ = .127, *d* = .38; *p*_T0-T2_ = .137, *d* = .39). There was also no difference between T1 and T2 (*p*_T1-T2_ = .876, *d* = .04). Thus, the trajectory was labeled as stable over time at a high level.

#### Sedentary behavior

LCGA suggested that there were two latent subgroups regarding trajectories in SB. The entropy for the two-class solution was slightly below .800, but the elbow plot (Additional file 3), likelihood ratio tests (Table 1) and classification probabilities (Table 2) provided sufficient evidence to select the two-class model.

The sample consisted of a “slightly decreasing high SB” class and a “slightly increasing moderate SB” class. There were *n* = 63 individuals in the “slightly decreasing high SB” class, with daily mean (*SE*) SB = 475.67 (9.68) minutes at T0, 444.28 (16.02) minutes at T1 and 437.09 (17.19) minutes at T2. Thus, at all timepoints the mean sedentary time was exceeding seven hours per day. Yet, there was a statistically significant mean decrease in SB between T0 and T2 by roughly 39 min with a small effect size (*p*_T0-T2_ = .022, *d* = .36). There was no significant difference between T0 and T1 (*p*_T0-T1_ = .073, *d* = .24) or between T1 and T2 (*p*_T1-T2_ = .720, *d* = .05).

The “slightly increasing moderate SB” class consisted of *n* = 152 individuals with daily mean (*SE*) SB = 263.38 (5.93) minutes at T0, 256.82 (9.82) minutes at T1 and 284.58 (8.56) minutes at T2. At each timepoint, the mean sedentary time equaled between four and five hours per day. SB did not differ significantly between T0 and T1 (*p*_T0-T1_ = .523, *d* = .08). It increased significantly by roughly 21 min between T0 and T2 (*p*_T0-T2_ = .019, *d* = .27). The difference between T1 and T2 was also significant (*p*_T1-T2_ = .008, *d* = .30). Yet, effect sizes were small.

### Changes within latent trajectories

**Fig. 1:**
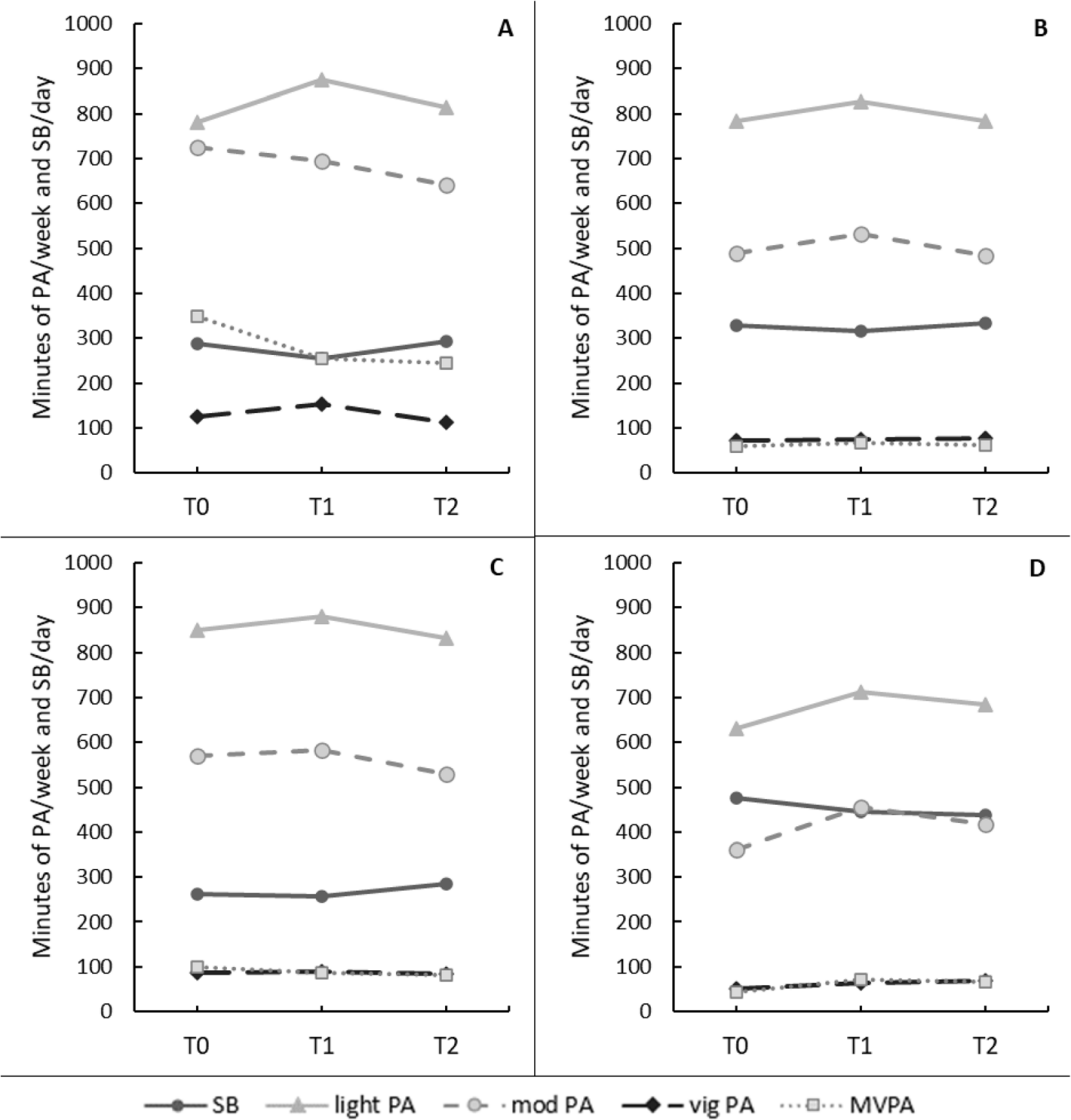
*Changes in Physical Activity and Sedentary Behavior Over the Study Period by Latent Trajectory Class. *Moderate-to-vigorous physical activity (MVPA) is lower than moderate and vigorous physical activity combined, because for MVPA calculation, only the time spent in bouts of at least ten minutes was counted. SB = sedentary behavior per day in 30-min bouts; PA = physical activity; mod = moderate; vig = vigorous; MVPA = moderate-to-vigorous physical activity in 10-min bouts. A. “stable high MVPA” class. B. “stable insufficient MVPA” class. C. “slightly increasing moderate SB” class. D. “slightly decreasing high SB” class

In the “stable high MVPA” class, light physical activity seemed to increase at T1, but the difference in minutes was not significant by T2, with a moderate effect size (*p*_T0-T2_ = .590, *d* = .59). There were no significant changes in SB, moderate or vigorous physical activity. These findings support the assumption of stability across the study period (see Fig. 1 A).

In the “stable insufficient MVPA” class, light physical activity increased significantly between T0 and T1 (*p*_T0-T1_ = .007, *d* = .22), but this effect did not last until T2 (*p*_T0-T2_ = .995, *d* = .001). The same pattern was observed for moderate physical activity. There were no significant changes in vigorous physical activity or SB. Thus, this trajectory was mainly characterized by stability over time (see Fig. 1 B).

In the “slightly increasing moderate SB” class, there were no significant changes in MVPA, light, or vigorous physical activity, and very small effect sizes were noted. Moderate physical activity seemed to increase slightly at T1 but decreased significantly at T2 (*p*_T0-T1_ = .363, *d* = .08; *p*_T1-T2_ = .011, *d* = .30; *p*_T0-T2_ = .035, *d* = .23). This finding supported the assumption that this subgroup experienced a negative trajectory over time (see Fig. 1 C).

For the “slightly decreasing high SB” class, findings supported the positive trajectory (see Fig. 1 D). With a small effect size, MVPA increased significantly at T1, but the difference did not remain significant at T2 (*p*_T0-T1_ = .024, *d* = .32; *p*_T0-T2_ = .120, *d* = .26). However, significant increases between T0 and T2 were observed in moderate (*p*_T0-T2_ = .011, *d* = .43), and vigorous physical activity (*p*_T0-T2_ = .003, *d* = .49).

### Associations between social-cognitive predictors and latent trajectories

Individuals in the “stable high MVPA” class reported significantly higher baseline levels of action planning compared to the “stable insufficient MVPA” class (*d* = .50). None of the other included baseline social-cognitive predictors were significantly associated with latent MVPA change trajectory class membership (see Table 3). Only action self-efficacy significantly predicted membership of the SB trajectories, with the “slightly decreasing high SB” class reporting higher baseline levels of action self-efficacy compared to the “slightly increasing moderate SB” class (*d* = .33, see Table 3).

**Table 3:** Associations of Latent Physical Activity and Sedentary Behavior Trajectory Classes with Baseline Characteristics

	Total *n* = 215	Moderate-to-vigorous physical activity			Sedentary Behavior		
		Stable high *n* = 18	Stable insuff. *n* = 197	Effect size	Increasing mod. *n* = 152	Decreasing high *n* = 63	Effect size
**Social-cognitive predictors:** ***M*** **(*SE*)**				**Cohen’s** ***d***			**Cohen’s** ***d***
POE	6.24 (0.08)	6.28 (0.24)	6.24 (0.09)	.03	6.20 (0.10)	6.35 (0.14)	.13
NOE - Takes too long	3.72 (0.18)	3.42 (0.55)	3.75 (0.19)	.16	3.67 (0.20)	3.84 (0.30)	.08
NOE - Too costly	2.38 (0.15)	1.92 (0.41)	2.42 (0.17)	.26	2.42 (0.19)	2.28 (0.27)	.08
Intention	5.25 (0.12)	5.29 (0.42)	5.25 (0.12)	.03	5.28 (0.14)	5.19 (0.21)	.05
Action S-E	4.88 (0.12)	5.11 (0.29)	4.86 (0.12)	.15	**4.72 (0.14)**	**5.27 (0.22)**	**.33**
Maintenance S-E	4.55 (0.10)	4.83 (0.31)	4.52 (0.11)	.20	4.48 (0.13)	4.71 (0.18)	.15
Recovery S-E	4.77 (0.11)	4.47 (0.33)	4.80 (0.12)	.21	4.65 (0.13)	5.08 (0.20)	.27
Action planning	4.49 (0.15)	**5.46 (0.43)**	**4.40 (0.16)**	**.50**	4.50 (0.17)	4.46 (0.19)	.02
Coping planning	3.81 (0.13)	4.03 (0.44)	3.79 (0.14)	.12	3.82 (0.16)	3.78 (0.25)	.02
Habit strength	3.03 (0.14)	3.92 (0.51)	2.94 (0.15)	.47	3.08 (0.17)	2.90 (0.26)	.08
**Continuous Covariates:** ***M*** **(*SE*)**							
Age	68.44 (0.36)	67.50 (1.08)	68.53 (0.38)	.19	68.14 (0.41)	69.17 (0.72)	.20
Wear-time (min/day)	832.23 (5.60)	816.26 (13.70)	833.69 (5.98)	.21	**814.89 (6.54)**	**874.06 (8.86)**	**.76**
**Categorical covariates:** ***n*** **(%)**				**Cramer’s** ***V***			**Cramer’s** ***V***
Sex, female	143 (66.5)	10 (55.6)	133 (67.5)	.07	**109 (71.7)**	**34 (54.0)**	**.17**
ISCED, high	118 (54.9)	12 (66.7)	106 (53.8)	.07	86 (56.6)	32 (50.8)	.05
Fully retired	124 (57.7)	9 (50.0)	115 (58.4)	.05	82 (53.9)	42 (66.7)	.12
Income				.09			.14
moderate	80 (37.2)	8 (44.4)	74 (37.6)		52 (34.2)	29 (46.0)	
high	72 (33.5)	7 (38.9)	63 (32.0)		56 (36.8)	15 (23.8)	
Overweight/obese	123 (57.2)	9 (50.0)	113 (57.4)	.04	**77 (50.7)**	**45 (71.4)**	**.19**
Group, web-based	121 (56.3)	12 (66.7)	109 (55.3)	.06	86 (56.6)	35 (55.6)	.01

*Note*. Associations were tested on a univariate level using Chi^2^- or T-tests. Statistically significant differences (*p*-value <.05) are in bold

*insuff.* insufficient, *ISCED* International Standard of Education, *M* (*SE*) mean (standard error), *mod.* moderate, *NOE* negative outcome expectations, *POE* positive outcome expectations, *S-E* self-efficacy

Reference categories: male sex; low/moderate ISCED; other than fully retired; low income; underweight/normal weight; print-based intervention group

## Discussion

The analyses reported in this study were conducted assuming that change in MVPA and SB occurs heterogeneously in a nine-month intervention study for the promotion of physical activity targeted at older adults. In line with this hypothesis, latent subgroups could be identified for each behavior: a “stable high MVPA” and a “stable insufficient MVPA” trajectory, as well as a “slightly decreasing high SB” and a “slightly increasing moderate SB” trajectory. Effect sizes were mostly small but might be clinically relevant. Contrary to the second hypothesis, social-cognitive variables at baseline were not significantly associated with the latent trajectories, except for action planning and action self-efficacy.

Individuals who were consistently sufficiently physically active during the study period had higher levels of action planning at baseline. Individuals who changed their behavior towards decreasing SB and increasing MVPA during the study period had higher levels of action self-efficacy at baseline. This finding matches theory [13–15]. Generally, physical activity interventions have been reported to be effective in older adults [7–9, 39]. This study’s assumption was that such a positive trajectory might be masked for the whole study sample in primary outcome analyses [38], but that it might occur in distinct latent subgroups. This phenomenon could be shown for individuals identified as belonging to the “slightly decreasing high SB” class, as they could significantly decrease sitting by approximately 40 min and increase moderate and vigorous physical activity by approximately 60 and 20 min, respectively. The latent MVPA trajectories, however, were both stable, as there was no significant change by the end of the study period, even though short-term increases may have been present at T1.

A potential explanation for the decline in physical activity after T1 could be the concurrent end of weekly group meetings which were only part of the first intervention phase. A longitudinal study has shown that exercise group membership predicted long-term physical activity engagement in older adults [40]. In a group-based randomized trial targeting older adults, exercise adherence was found to be associated with perceived group cohesion [41]. Several other findings reported here are corroborated by former studies. For example, a high prevalence of sitting for more than four hours per day is in line with previous research on SB in older adults [42]. According to a systematic review on physical activity trajectories during the life course, the inactivity trajectories seem to be more stable than the activity trajectories [21] which highlights the difficulty of promoting a behavior change in old age. This finding might also explain why an “increasing MVPA” trajectory could not be identified in this sample of rather inactive older adults. In fact, there was a low proportion of very active older adults already at baseline. As the inclusion criteria allowed older adults to be either initially inactive or recently active, it is possible that the highly active individuals had started to be sufficiently physically active within the past year and were successful in turning their activity into a habit during the study period.

### Study strengths and limitations

The existence of three assessment points enabled the calculation of statistically advanced longitudinal mixture models, granting novel insights into latent trajectories in a physical activity intervention study targeted at older adults. The physical activity data were objectively assessed, which is an advantage over many studies assessing self-reported data.

However, the results of this study need to be interpreted carefully, acknowledging various methodological weaknesses. Only linear trends could be investigated as there were only three timepoints to be considered. Alternative functions, such as quadratic or cubic functions, might have fit the data better, but testing this was not possible as this requires more than three timepoints. Another major limitation of this study is its sample size and a missing power calculation, as the analyses presented here are only exploratory in nature. This has been described as a common concern in LCGA performed on intervention data [22]. Even though the selected finite mixture models converged successfully and provided theoretically and statistically plausible as well as meaningful results, the subgroup analyses suffered from low cell counts. It could therefore be argued that assessing the clinical utility of the observed model structure requires a larger sample size, even though it met the criterium of a minimum sample size of 200 participants [43]. Also, this study used a classify-analyze approach, which is criticized for not addressing the classification uncertainty when analyzing predictors of latent class membership [44]. However, this criticism was based on cross-sectional and not developmental latent class analysis. Adding the social-cognitive predictors to the LCGA model, which is the suggested solution for cross-sectional latent class analyses, was not feasible as any missing baseline values in social-cognitive predictors would have significantly reduced the analysis sample size.

It also needs to be noted that assessments took place in different seasons: T0 and T2 in fall/winter months and T1 in spring/summer months. The physical activity level of German community-dwelling older adults has been shown to be influenced by weather conditions on a cross-sectional level [45, 46]. However, this association may have less relevance in longitudinal within-person changes. In latent class growth models testing within-person changes in steps in a sample of women, for example, seasonal changes did not seem to account for a practically significant difference [47]. A recent study of German young and middle-aged adults has shown high variability in wearable usage between individuals, but non-significant main effects for weather conditions, suggesting that these external factors may be less relevant than individual factors in continuous use [48]. Yet, it cannot be ruled out that increases in physical activity level at T1 were related to the assessment having taken place in spring or summer. Furthermore, social-cognitive predictors of behavior change were only assessed for physical activity as this was the intervention trial’s target behavior, but not for sitting. Associations between SB trajectory class and social-cognitive predictors might, therefore, have been weak. These analyses could also be limited because they did not include the latest definition of MVPA according to the World Health Organization. They recently adapted their physical activity recommendations with the most significant modification being the removal of the ten-minute bout benchmark for MVPA [49, 50]. This study, however, considered MVPA in bouts of at least ten minutes as the primary outcome variable, as this was the recommendation given to study participants. Lastly, the external validity of this study is limited in terms of the results stemming from a selected sample of older adults matching the rather strict inclusion criteria, that is, access to mobile technology, and absence of cognitive or health impairments.

## Conclusions

Identifying short-term physical activity trajectories in intervention studies can provide valuable insights on the change patterns in heterogenous study samples. Knowledge regarding baseline social-cognitive indicators associated with latent MVPA and SB trajectories (such as action self-efficacy and action planning) could be used in future research to better address the needs of particular latent trajectory classes. Theories on health behavior change may be utilized to identify distinct needs. However, research in this field is scarce and the advanced analyses require longitudinal studies with high methodological quality, including sufficiently long follow-up periods, repeated measurements, and a sufficient sample size.

This study contributes to the research field of longitudinal health behavior change by suggesting a more tailored approach. Promoting an increasing change trajectory in initially inactive older adults requires large efforts and calls for targeted intervention strategies. Findings propose that certain characteristics may serve as predictors of latent change trajectories and that researching this further can unveil distinct needs of inactive individuals who are likely to belong to stable or decreasing change trajectories.

## Supplementary Information

**Additional file 1 ***Flow Chart.*

**Additional file 2 ***Elbow Plot of Fit Statistics Based on Latent Class Growth Analysis of Physical Activity Change Trajectories****.*** The point at which the information criterion value becomes stable, even if more classes are added, is used as indication of the solution best fitting the data. AIC = Akaike’s information criterion; BIC = Bayesian information criterion; SABIC = sample size adjusted BIC.

**Additional file 3 ***Elbow Plot of Fit Statistics Based on Latent Class Growth Analysis of Sedentary Behavior Change Trajectories. *The point at which the information criterion value becomes stable, even if more classes are added, is used as indication of the solution best fitting the data. AIC = Akaike’s information criterion; BIC = Bayesian information criterion; SABIC = sample size adjusted BIC.

## Data Availability

The dataset analyzed during the current study is not publicly available as it contains indirect identifiers and consent for its publication could not be obtained, as this would violate confidentiality. The analyzed dataset is available from the corresponding author on reasonable request.

## Abbreviations

AIC: Akaike’s information criterion
BIC: Bayesian information criterion
BLRT: Bootstrapped likelihood ratio test
GRoLTS: Guidelines for reporting on latent trajectory studies
HAPA: Health action process approach
ISCED: International standard of education
LCGA: Latent class growth analysis
LMR-LRT: Lo-Mendell-Rubin likelihood ratio test
MMSE-2-BV: Mini mental state examination 2 - brief version
MVPA: Moderate-to-vigorous physical activity
SABIC: Sample-size adjusted Bayesian information criterion
SB: Sedentary behavior
SE: Standard error
