# Supplementary material for: Distinct physical activity and sedentary behavior trajectories in older adults during participation in a physical activity intervention: a latent class growth analysis
Source: Eur Rev Aging Phys Act. 2022 Jan 5;19:1. doi: 10.1186/s11556-021-00281-x (PMC8903622; doi:10.1186/s11556-021-00281-x)
Supplement: Supplementary file 1 — Additional file 1 Flow Chart. [file 11556_2021_281_MOESM1_ESM.docx]

**Additional file 1**

*Flow Chart*
