# Supplementary material for: Distinct physical activity and sedentary behavior trajectories in older adults during participation in a physical activity intervention: a latent class growth analysis
Source: Eur Rev Aging Phys Act. 2022 Jan 5;19:1. doi: 10.1186/s11556-021-00281-x (PMC8903622; doi:10.1186/s11556-021-00281-x)
Supplement: Supplementary file 2 — Additional file 2 Elbow Plot of Fit Statistics Based on Latent Class Growth Analysis of Physical Activity Change Trajectories. The point at which the information criterion value becomes stable, even if more classes are added, is used as indication of the solution best fitting the data. AIC = Akaike’s information criterion; BIC = Bayesian information criterion; SABIC = sample size adjusted BIC. [file 11556_2021_281_MOESM2_ESM.docx]

**Additional file 2**

*Elbow Plot of Fit Statistics Based on Latent Class Growth Analysis of Physical Activity Change Trajectories*

**
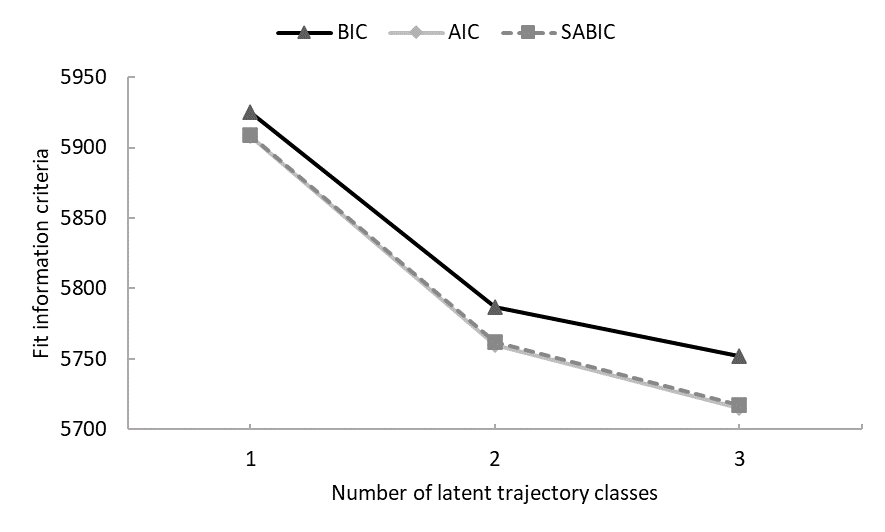
**

*Note.* The point at which the information criterion value becomes stable, even if more classes are added, is used as indication of the solution best fitting the data. AIC = Akaike’s information criterion; BIC = Bayesian information criterion; SABIC = sample size adjusted BIC**.**
